# Darifenacin: a promising chitinase 3-like 1 inhibitor to tackle drug resistance in pancreatic ductal adenocarcinoma

**DOI:** 10.1007/s00280-024-04712-1

**Published:** 2024-09-03

**Authors:** Sofia M. Sousa, Helena Branco, Amir Avan, Andreia Palmeira, Luca Morelli, Lúcio L. Santos, Elisa Giovannetti, M. Helena Vasconcelos, Cristina P. R. Xavier

**Affiliations:** 1 i3S - Instituto de Investigação e Inovação em Saúde, Universidade do Porto, Rua Alfredo Allen 208, Porto, 4200-135 Portugal; 2 Cancer Drug Resistance Group, IPATIMUP - Institute of Molecular Pathology and Immunology, University of Porto, Rua Alfredo Allen 208, Porto, 4200-135 Portugal; 3 LQOF – Laboratory of Organic and Pharmaceutical Chemistry, Department of Chemical Sciences, Faculty of Pharmacy, University of Porto, Rua de Jorge Viterbo Ferreira 228, Porto, 4050-313 Portugal; 4 Metabolic Syndrome Research Center, Mashhad University of Medical Sciences, Mashhad, 91886-17871 Iran; 5 Medical Genetics Research Center, Faculty of Medicine, Mashhad University of Medical Sciences, Mashhad, 91886-17871 Iran; 6 CIIMAR – Interdisciplinary Centre of Marine and Environmental Research, Terminal de Cruzeiros do Porto de Leixões, Matosinhos, 4450-208 Portugal; 7 General Surgery Unit, Department of Translational Research and New Technologies in Medicine and Surgery, University of Pisa, Pisa, 56100 Italy; 8 Experimental Pathology and Therapeutics Research Group and Surgical Oncology Department, IPO—Instituto Português de Oncologia, Rua Dr. António Bernardino de Almeida 865, Porto, 4200-072 Portugal; 9 ICBAS-UP—School of Medicine and Biomedical Sciences, University of Porto, Rua de Jorge Viterbo Ferreira 228, Porto, 4050- 313 Portugal; 10 Department of Medical Oncology, Cancer Center Amsterdam, Amsterdam UMC, Vrije Universiteit, HV Amsterdam, 1081 The Netherlands; 11 Cancer Pharmacology Lab, Fondazione Pisana per La Scienza, San Giuliano, 56017 Italy; 12 Department of Biological Sciences, FFUP – Faculty of Pharmacy, University of Porto, Rua de Jorge Viterbo Ferreira 228, Porto, 4050-313 Portugal; 13 UCIBIO - Applied Molecular Biosciences Unit, Toxicologic Pathology Research Laboratory, University Institute of Health Sciences (1H-TOXRUN, IUCS-CESPU), Gandra, 4585-116 Portugal; 14 Associate Laboratory i4HB - Institute for Health and Bioeconomy, University Institute of Health Sciences - CESPU, Gandra, 4585-116 Portugal

**Keywords:** Cancer drug resistance, Cancer treatment, Chitinase-3 like 1, Darifenacin, Molecular docking, Pancreatic cancer

## Abstract

**Purpose:**

Pancreatic ductal adenocarcinoma (PDAC) is among the most aggressive malignancies. Our previous work revealed Chitinase 3-like 1 (CHI3L1) involvement in PDAC resistance to gemcitabine, identifying it as a promising therapeutic target. Here, we aimed to identify putative CHI3L1 inhibitors and to investigate their chemosensitizing potential in PDAC.

**Methods:**

Docking analysis for CHI3L1 identified promising CHI3L1 inhibitors, including darifenacin (muscarinic receptor antagonist). PDAC cell lines (BxPC-3, PANC-1) and primary PDAC cells were used to evaluate darifenacin’s effects on cell growth (Sulforhodamine B, SRB), alone or in combination with gemcitabine or gemcitabine plus paclitaxel. Cytotoxicity against normal immortalized pancreatic ductal cells (HPNE) was assessed. Recombinant protein was used to confirm the impact of darifenacin on CHI3L1-induced PDAC cellular resistance to therapy (SRB assay). Darifenacin’s effect on Akt activation was analysed by ELISA. The association between cholinergic receptor muscarinic 3 (CHRM3) expression and therapeutic response was evaluated by immunohistochemistry of paraffin-embedded tissues from surgical resections of a 68 patients’ cohort.

**Results:**

*In silico* screening revealed the ability of darifenacin to target CHI3L1 with high efficiency. Darifenacin inhibited PDAC cell growth, with a GI_50_ of 26 and 13.6 µM in BxPC-3 and PANC-1 cells, respectively. These results were confirmed in primary PDAC-3 cells, while darifenacin showed no cytotoxicity against HPNE cells. Importantly, darifenacin sensitized PDAC cells to standard chemotherapies, reverted CHI3L1-induced PDAC cellular resistance to therapy, and decreased Akt phosphorylation. Additionally, high CHMR3 expression was associated with low therapeutic response to gemcitabine.

**Conclusion:**

This work highlights the potential of darifenacin as a chemosensitizer for PDAC treatment.

**Supplementary Information:**

The online version contains supplementary material available at 10.1007/s00280-024-04712-1.

## Introduction

Pancreatic cancer stands as the 3rd most frequent cause of cancer-associated mortality worldwide and its incidence is estimated to increase, with approximately 64,050 new cases predicted in 2023 [1]. Pancreatic ductal adenocarcinoma (PDAC) represents more than 90% of all diagnosed cases [2], with a 5-year survival rate of approximately 12% [1, 3]. Unfortunately, PDAC is considered a notoriously silently growing tumor, with almost no visible or distinctive symptoms at an early stage, and with only around 20% of patients being eligible for radical surgery [3]. Owing to the dense desmoplastic stroma, heterogeneity of genetic mutations, multiple signaling pathway alterations and metastatic potential, PDAC is considered one of the most chemoresistant types of cancer [4–6]. Regarding treatment, gemcitabine is administered alone or in combination with other chemotherapeutic agents, namely paclitaxel [7]. The administration of FOLFIRINOX, a combination regimen of 5-fluorouracil/leucovorin with irinotecan and oxaliplatin, is also an option for PDAC patients, but its use remains limited due to its toxicological and adverse health effects [8].

Recently, our group identified chitinase 3-like-1 protein (CHI3L1) as an encouraging molecular target, impacting the therapeutic response of PDAC cells to gemcitabine treatment [9]. Other studies also reported an association between high expression levels of CHI3L1 and low survival, poor prognosis or advanced tumor stage in patients with PDAC [10, 11]. Moreover, CHI3L1 is also upregulated in several inflammation-related diseases [12, 13]. CHI3L1 is a secreted glycoprotein that has the ability to bind to chitin-like oligosaccharides of different lengths; however, it lacks chitinase activity due to the substitution of an essential glutamic acid with leucine in the catalytic domain. CHI3L1 also interacts with other molecules, including heparin, hyaluronic acid, and all three forms of collagen (types I, II and III) [14]. This protein is also named YKL-40, given the molecular weight of 40 kDa and the three N-terminal amino acid residues present in the secreted form: tyrosine (Y), lysine (K) and leucine (L) [15]. The CHI3L1 protein consists of a single polypeptide chain of 383 amino acids [16] and is characterized by an isoelectric point of 7.6 [17]. Studies of the three-dimensional (3D) structure of CHI3L1 showed a division into two globular domains: an eight-stranded β/α-barrel domain with a TIM fold and a second domain composed of six antiparallel β-strands, with one α-helix (α + β) domain inserted after strand β7 [18]. Furthermore, a 43-Å long carbohydrate-binding groove was discovered at the C-terminal side of the β-strands in the (β/α)_8_-barrel, where the protein-carbohydrate interactions are dominated by the stacking of aromatic amino acid residue side chains [19, 20].

Given our previous work that showed the impact of CHI3L1 on PDAC therapeutic response, the investigation of therapeutic modalities capable of targeting CHI3L1 in an adjuvant setting would be of great interest. Indeed, there are numerous repurposed drug candidates that may target CHI3L1, and that could be used in combination with conventional chemotherapy to broaden therapeutic options and provide a faster response to PDAC treatment [21]. Therefore, our work aimed to: (i) identify, through a molecular docking *in silico* study, known drugs from the DrugBank database with the ability to bind CHI3L1; and to (ii) confirm the sensitizing effect of darifenacin, one of the CHI3L1 inhibitors identified with good docking scores, to chemotherapeutic drugs commonly used in PDAC treatment.

## Methods

### Receptor and ligands for virtual screening

Using the Protein Data Bank (pdb code: 1NWU), the 3D structure of CHI3L1 at a resolution of 2.20 Å was acquired. The protein target was prepared using AutoDock Tools 1.5.6 (Scripps Research Institute, CA, USA) [22]. The 3D structures of the controls were drawn using ChemDraw 15.1 and minimized by the MM2 force field method at a root mean square deviation (RMSD) gradient of 0.01 kcal/mol.Å using Chem3D 15.1 (PerkinElmer, Diamond, USA). A total of 11,741 molecules (approved and experimental drugs, compounds under investigation, nutraceuticals, illicit and withdrawn compounds) were taken from the DrugBank database (Version 5.1.8) [23]. The energy minimization of all database was accomplished using the MM2 force field method at an RMSD gradient of 0.01 kcal/mol.Å. The original chirality was maintained, and partial charges were calculated based on the standard parameters of the force field.

### Molecular docking virtual screening for chitinase 3-like-1 (CHI3L1)

Docking simulations between the CHI3L1 (pdb code: 1NWU) and the controls and database molecules were undertaken using AutoDock Vina (Scripps Research Institute) and the program PyRx 0.8, which allows an automated screen [24]. For this analysis, the target conformation was considered a rigid unit, and the ligands were flexible and adaptable to the target. AutoDock Vina looked for the minimal binding energy conformations and sent nine different conformations for each ligand. It was run using a thoroughness of eight and a grid box with the proportions of 19.35, 27.08, and 24.07 Å built around the crystallographic ligand. Conformations and interactions were visualized using PyMOL version 2.4 (Schrödinger) [25, 26].

### Chemicals

Darifenacin hydrobromide (darifenacin; SML1102), gemcitabine (G6423) and paclitaxel (T7402), all from Merck Life Science, were dissolved in dimethyl sulfoxide (DMSO) and pentoxifylline (P1784, Merck Life Science) in sterile water. All compounds were stored at − 20 ºC. Recombinant human (rh) protein chitinase 3-like-1 (rhCHI3L1; 2599-CH, R&D Systems) was dissolved in filtered phosphate-buffered saline (PBS) at a concentration of 200 µg/mL.

### Cell culture of cell lines

BxPC-3 and PANC-1 human pancreatic cancer cell lines were grown in Dulbecco’s Modified Eagle Medium (DMEM) supplemented with 4.5 g/L Glucose and UltraGlutamine^™^ w/sodium pyruvate (Lonza) complemented with 10% fetal bovine serum (FBS, Biowest). The human pancreatic duct epithelial-like cell line hTERT-HPNE (HPNE) was obtained from the American Type Culture Collection (ATCC) and cultured in DMEM medium (Sigma) supplemented with 10% fetal calf serum (FCS) and 10 ng/mL human recombinant epidermal growth factor (Sigma). The non-tumorigenic human breast epithelial cell line MCF-10 A was maintained in DMEM/F12 (Thermo Fischer Scientific), supplemented with 5% inactivated Horse Serum (HS), 0.5 mg/mL of hydrocortisone, 20 ng/mL human epidermal growth factor, 10 mg/mL of insulin, 100 ng/mL of cholera toxin, 100 units/mL penicillin and 100 mg/mL of streptomycin, as previously described [27]. Cells were maintained in a humidified atmosphere supplied with 5% CO2 at 37 °C. Cell number and viability were evaluated using the Trypan Blue exclusion assay. All experiments were performed with cells at the exponential phase of growth and having more than 90% viability. For the sulforhodamine B assay, cells were grown in medium supplemented with 5% FBS. All cell lines were genotyped and routinely tested for Mycoplasma contamination.

### Primary cell culture

PDAC-3 primary human PDAC cells were acquired from resected patients, as previously explained, in agreement with the 1975 Declaration of Helsinki [28]. Cells were grown in Roswell Park Memorial Institute medium (RPMI, Lonza) supplemented with 10% FCS (Biowest) and 1% penicillin/streptomycin (Lonza) and grown at 37 °C in a humidified chamber at 5% CO2. Cells were monthly tested for mycoplasma contamination using the MycoAlert Myco-plasma Detection kit (Westburg).

### Drug treatments

To determine the concentration that inhibits 50% of cell growth (GI_50_) of each individual drug, BxPC-3 and PANC-1 cell lines were treated for 48 h with: (a) five serial dilutions of the tested drugs; (b) vehicle (at the highest concentration used); and (c) medium only. Darifenacin was tested at concentrations varying from 6.25 µM to 100 µM and paclitaxel from 1.88 to 30 nM or 9.38 to 150 nM, for the PANC-1 or BxPC-3 cells, respectively. A similar range of concentration (from 0.1 to 100 µM) was used to study the effects of darifenacin in PDAC-3 primary cells and on the HPNE cells.

The effect of CHI3L1 on PDAC cell growth following drug treatments was also assessed. For that, BxPC-3 and PANC-1 cells were cultured with rhCHI3L1 at 250 or 1000 ng/mL, respectively. With regard to BxPC-3 cells, after a 16 h incubation period, cells were treated for 48 h with: (a) gemcitabine at 51 nM and paclitaxel at 2 nM; (b) vehicle (at the highest concentration tested); and (c) medium alone. Accordingly, PANC-1 cells were treated for 48 h with: (i) darifenacin at 10 µM; (ii) gemcitabine at 83 nM and paclitaxel at 20 nM; (iii) gemcitabine at 83 nM, paclitaxel at 20 nM and darifenacin at 10 µM; (iv) vehicle (at the highest concentration tested); and (v) medium alone.

### Cell growth determination using the sulforhodamine B (SRB) assay

The SRB assay allows to understand the cell growth inhibitory effect, as previously described [9, 29]. BxPC-3 (7.5 × 10^4^ cells/mL) and PANC-1 (5.0 × 10^4^ cells/mL) cells were seeded in 96-well plates in a total volume of 100 µL/well [9]. MCF-10 A cells were seeded at a concentration of 5.0 × 10^4^ cells/mL [27]. Regarding PDAC-3 primary cells, 3.0 × 10^4^ cells/mL were seeded, while HPNE cells were seeded at a concentration of 8.0 × 10^4^ cells/mL. Following incubations with compounds and control treatments, cells were fixed with 10% (w/v) ice-cold trichloroacetic acid (TCA), washed with distilled water and stained with 0.4% (w/v) SRB (Merck Life Science) prepared in 1% acetic acid. Next, cells were washed with 1% (v/v) acetic acid, and the SRB solubilized using 10 mM Tris base solution. The absorbance was measured in a multiplate reader (Synergy^™^ Mx, Biotek Instruments Inc.) [30].

### Phospho-Akt and total Akt evaluation by enzyme linked immunosorbent (ELISA) assay

Akt phosphorylation at serine residue 473 (pS473Akt) and total Akt content were analyzed using AKT Colorimetric ELISA kits (Thermo Scientific). PANC-1 cells were seeded in a 96-well plate (1 × 10^5^ cells per well) and treated for 24 h with darifenacin at 10 µM. The phospho-Akt and total Akt levels were assessed, as described previously [31].

### Evaluation of cholinergic receptor muscarinic 3 (CHRM3) protein expression by immunohistochemistry (IHC) in PDAC patients

The expression levels of Cholinergic Receptor Muscarinic 3 (CHRM3) was assessed by immunohistochemistry (IHC) in paraffin-embedded tissues obtained during surgical resections in a cohort of 68 patients who underwent pancreatectomies at the University Hospital of Pisa (Pisa, Italy) and at the Mashhad University Hospital (Mashhad, Iran), as described previously [32]. The procedures for handling human materials were performed in accordance with the 1975 Declaration of Helsinki. All specimens were acquired after the patient’s written consent approved by the local Ethics Committees, as described previously [33]. Before staining, the tissue slides were deparaffinized using xylene and rehydrated in ethanol. Immunostaining was performed using the avidin–biotin DAKO EnVision^™^ peroxidase complex method (Dako). The slides were placed in a 95 °C solution of 0.01 M sodium citrate buffer (pH 6.0) for antigen retrieval. The staining with the rabbit anti-M3 polyclonal antibody (dilution, 1:200; Santa Cruz Biotechnology) was applied overnight at 4 °C. Immunostaining intensity was classified from 0 to 4+, and considering both positive cells’ number and intensity. Thus, 0 means no staining, 1 + means focal weak staining, 2 + means focal strong staining or diffuse weak staining, 3 + means diffuse medium staining and 4 + means diffuse strong staining [34]. The median was chosen as a cut-off. Samples was “high CHRM3” when the staining score was > the median, and “low CHRM3” if the staining score was ≤ the median. Clinicopathological data of patients were acquired from electronic patient records (Supplementary Table 1), and survival data were verified from regional registries. The overall survival (OS) was determined from the time of diagnosis to death or last follow-up. Kaplan–Meier curves were generated, and OS evaluated by log-rank test.

### Statistical analysis

Statistical analyses were performed using GraphPad Prism V7.0 software, and data were analyzed with the student’s *t*-test. Statistical significance was set at *p* < 0.05. Data are expressed as mean ± standard error of the mean (S.E.M.) of at least three independent experiments. Regarding the correlation of C*HRM3* with survival, data were analyzed using SPSS^®^ Statistics version 23 statistical software (IBM, Chicago, IL, USA) and GraphPad Prism V7.0 software was used to create plots. Statistical significance was set at *p* < 0.05.

## Results

### Virtual screening for CHI3L1

**Fig. 1:**
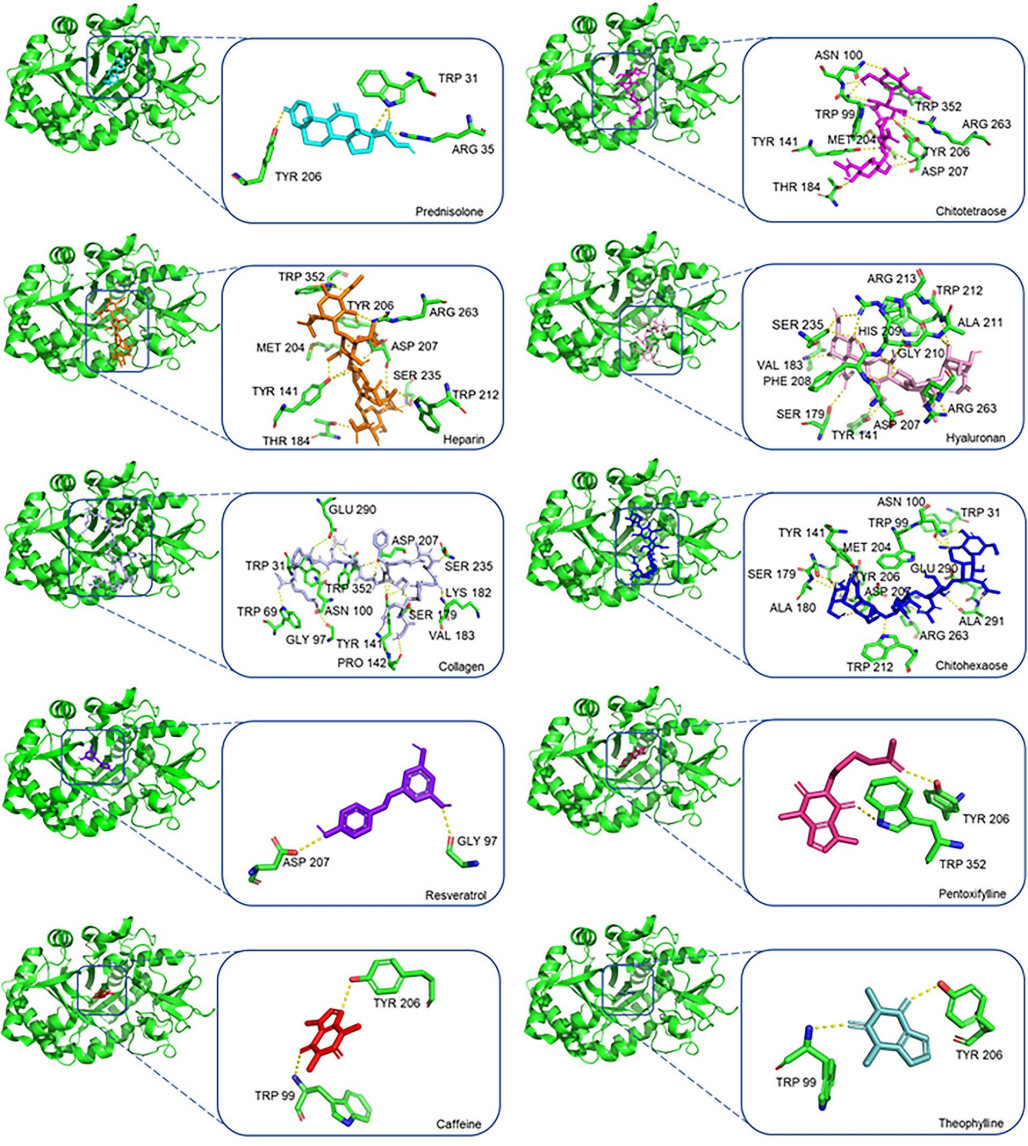
Visual representation of positive control docking conformations and its interactions with CHI3L1. CHI3L1 is represented as a ribbon, whereas the positive controls are represented as sticks. Hydrogen bond interactions are represented as yellow broken lines and the residues involved in such interactions are depicted as green sticks and labelled

Docking studies were performed on the 2.20 Å structure of *Homo sapiens* CHI3L1 (pdb code: 1NWU), using a series of known compounds described in the literature, which include ligands (chitotetraose [35], chitohexaose [36], heparin [14, 36], collagen [14, 36] and hyaluronan [36]) and CHI3L1 inhibitors (caffeine [37], pentoxifylline [9], prednisolone [38], resveratrol [38, 39], theophylline [37]). The positive controls revealed scoring values between − 5.9 and − 10.1 kcal/mol. Amongst them, the inhibitor prednisolone was shown to bind to CHI3L1 with the highest affinity, presenting the most negative docking score value (-10.1 kcal/mol). A visual interpretation of the conformation and interaction of prednisolone and the other positive controls with CHI3L1 was performed to analyze the binding mechanism (Fig. 1). The compounds are ordered according to the degree of affinity to CHI3L1, i.e., from the most negative to the less negative value of score (-10.1 to -5.9 kcal/mol).

**Fig. 2:**
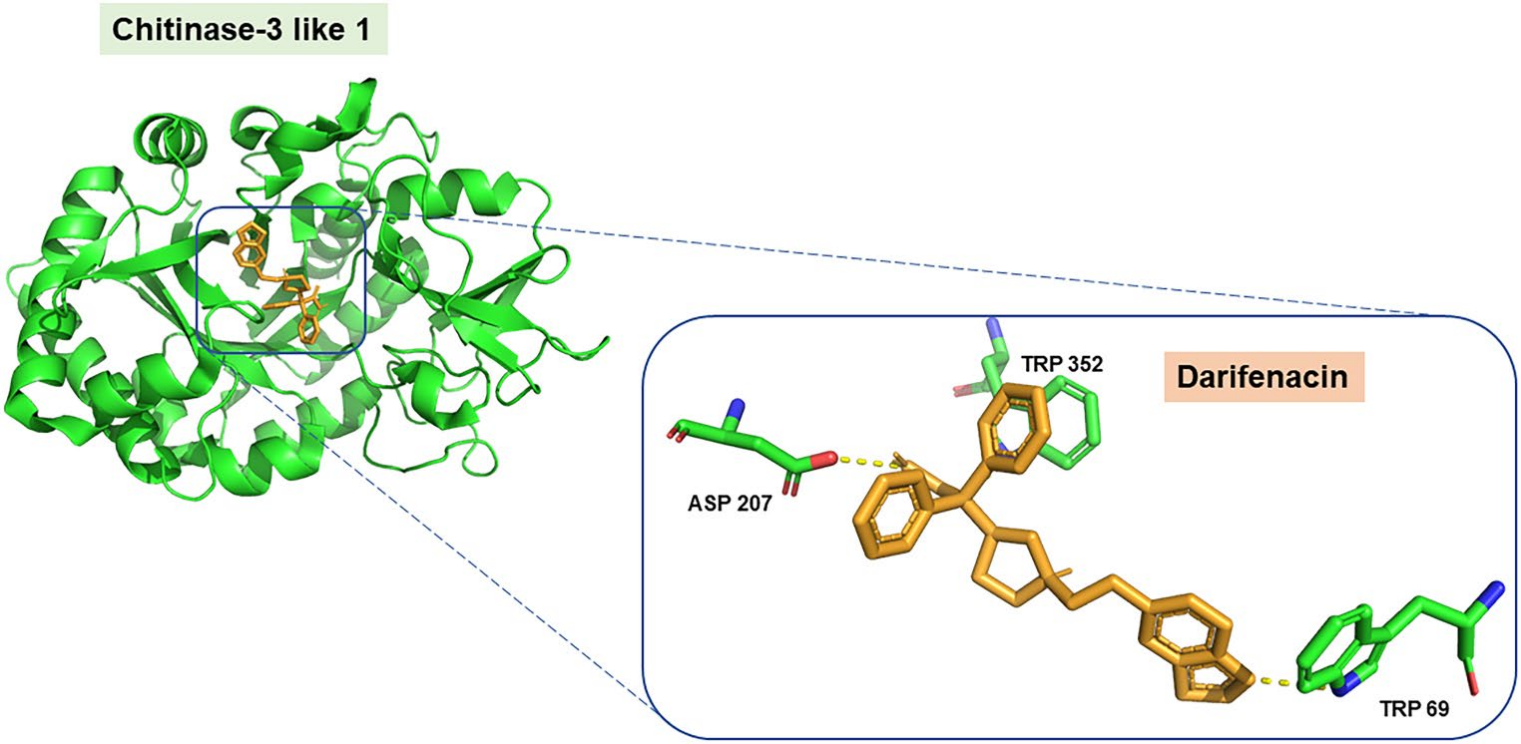
Visual representation of the conformation and interactions of darifenacin with CHI3L1. CHI3L1 is represented as a ribbon, whereas darifenacin is represented as orange sticks (hydrogen bond interactions in yellow broken lines, residues involved in the interaction in green sticks and labelled)

Then, 11,741 molecules from the DrugBank database were docked using the same methodology as for the positive controls. A total of 568 molecules (4.8% of the screened molecules) showed higher affinity for CHI3L1 than the inhibitor prednisolone, which is the positive control with the best score value (-10.1 kcal/mol). The drug pentoxifylline, approved for peripheral arterial diseases and a known inhibitor of CHI3L1 [40], also appeared as a positive control with a score value of -7.1 (Table 1). Indeed, we have reported that pentoxifylline reverts gemcitabine resistance induced by the recombinant protein of CHI3L1 in PDAC cells [9]. Most interestingly, the muscarinic receptor antagonist darifenacin showed a score value of -11.8 kcal/mol, which was lower than that of the well-known inhibitor prednisolone, suggesting that this drug could potentially target CHI3L1 with high efficiency (Table 1). Since darifenacin was at the top of the ranking of compounds, this drug was selected for further analysis of the interaction profile with the binding pocket of CHI3L1 (Fig. 2).

**Table 1:** Score values of darifenacin and positive controls

Compound	Score (kcal/mol)	Molecular Weight	logP	Therapeutic class
Darifenacin	-11.8	426.6	4.35	Muscarinic Receptor Antagonist
Prednisolone	-10.1	360.4	1.66	Glucocorticoid
Chitotetraose	-9.1	662.7	0.22	Chitin-like Oligosaccharides
Heparin	-8.8	1134.9	-13.20	Anticoagulant
Hyaluronan	-8.5	776.6	-1.80	Glycosaminoglycan
Collagen	-8.4	1471.6	-7.00	Protein
Chitohexaose	-7.9	985	-0.56	Chitin-like Oligosaccharides
Resveratrol	-7.5	228	2.57	Stilbenoid
Pentoxifylline	-7.1	278	0.08	Hemorheologic Agent
Caffeine	-5.9	194	-0.24	Stimulant
Theophylline	-5.9	180	-0.26	Phosphodiesterase Inhibitor

Further analysis was performed using the Protein-Ligand Interaction Profiler (PLIP) in order to identify other non-covalent interactions between CHI3L1 and the ligands (positive controls and darifenacin), such as hydrophobic interactions, π-cation interactions, salt bridges and π-stacking (Table 2).

**Table 2:** Interactions between darifenacin and the positive controls with the target CHI3L1, as predicted by the docking study

Compound	Hydrophobic Interactions	Hydrogen Bonds	π-Cation Interactions			Salt Bridges		π-Stacking
Darifenacin	TRP 31; PHE 58 TRP 69; TRP 99 ASN 100; LEU 140	TRP 69; ASP 207; TRP 352	---	---			TRP 31 TRP 352	
Prednisolone	TRP 31; TRP 99 TYR 206; PHE 261 THR 293; TRP 352	TRP 31; ARG 35; TYR 206	---		---			TRP 352
Chitotetraose	TYR 27; TRP 99 ALA 177; PHE 261 THR 293; TRP 352	TRP 99; ASN 100 TYR 141; THR 184 MET 204; TYR 206 ASP 207; ARG 263 TRP 352	---		---			---
Heparin	PHE 261 THR 293 LEU 356	TYR 141; THR 184 MET 204; TYR 206 ASP 207; TRP 212 SER 235; ARG 263 TRP 352	---		ARG 263			---
Hyaluronan	---	TYR 141; SER 179 VAL 183; ASP 207 PHE 208; HIS 209 GLY 210; ALA 211 TRP 212; ARG 213 SER 235; ARG 263	---		---			---
Collagen	TRP 31 PHE 58 TRP 69 TRP 99 TYR 141 PHE 208 TRP 212	TRP 31; TRP 69 GLY 97; ASN 100 TYR 141; PRO 142 SER 179; LYS 182 VAL 183; ASP 207 SER 235; GLU 290 TRP 352	ARG 263		---			ARG 263
Chitohexaose	TYR 141 MET 204 PRO 281	TRP 31; TRP 99 ASN 100; TYR 141 SER 179; ALA 180 MET 204; TYR 206 ASP 207; TRP 212 ARG 263; GLU 290 ALA 291	---		---			---
Resveratrol	TRP 31; PHE 58 TRP 69; TRP 99 ASN 100; TRP 352	GLY 97; ASP 207	---		---			---
Pentoxifylline	PHE 58; TRP 99 TRP 352	TYR 206; TRP 352	---		GLU 290			---
Caffeine	---	TRP 99; TYR 206	TRP 352		---			---
Theophylline	---	TRP 99; TYR 206	---		---			TRP 352

Therefore, taking into consideration the positive controls, our *in silico* molecular analysis identified darifenacin, an approved drug for the treatment of overactive bladder and urinary incontinence and an antagonist of the muscarinic M3 receptor, CHRM3 [41], with the ability to bind to CHI3L1, and thus with potential to have antitumor activity.

### Cell growth inhibitory effect of darifenacin in PDAC cell lines

The effect of darifenacin on PDAC cell growth was evaluated using the SRB colorimetric assay [30]. For that, two different human PDAC cell lines, BxPC-3 (wild-type *KRAS*) and PANC-1 (mutated *KRAS*) were used. The GI_50_ concentration (that causes 50% of cell growth inhibition) was determined. Our results demonstrated that darifenacin efficiently inhibited PDAC cell growth with GI_50_ concentrations of 26.0 ± 2.1 µM and 13.6 ± 2.7 µM for BxPC-3 and PANC-1 cells, respectively (Table 3).

**Table 3:** Determination of the GI_50_ concentrations of darifenacin in two PDAC cell lines

	Darifenacin
PDAC cell lines	**GI**_**50**_ (µM) ± S.E.M.*
BxPC-3	26.0 ± 2.1
PANC-1	13.6 ± 2.7

* Data shows the mean ± S.E.M. from at least three independent experiments

### Cell growth inhibitory effect of darifenacin in PDAC-3 primary culture cells

**Fig. 3:**
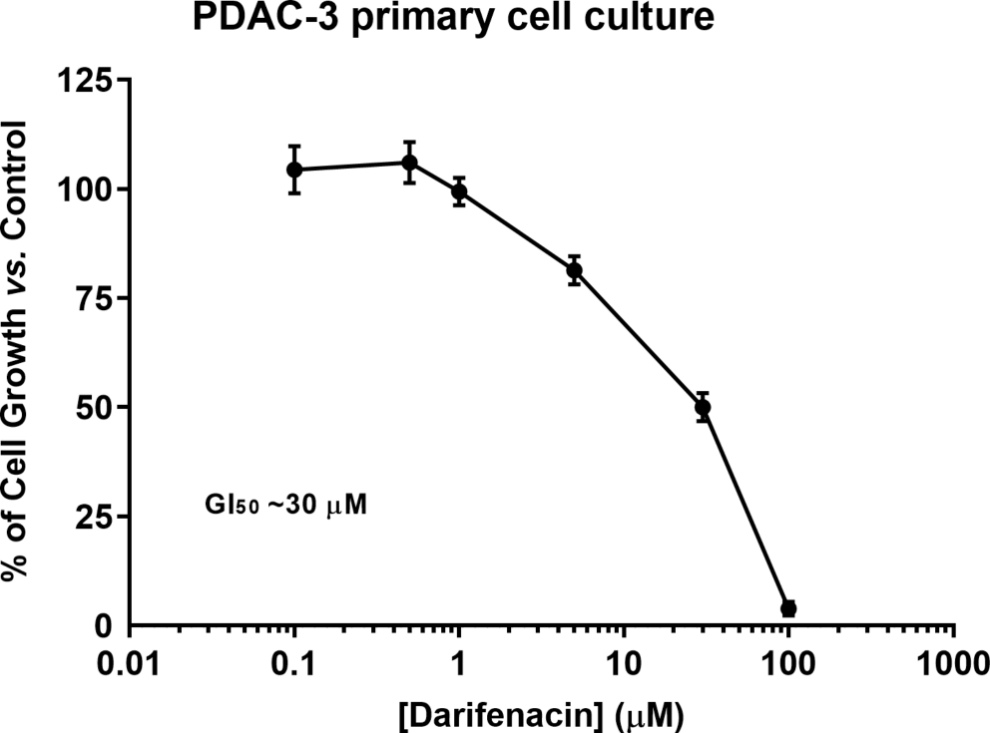
Dose-response curve of PDAC-3 primary culture cells following treatment with darifenacin. The percentage (%) of growth of the cells treated with darifenacin, at various concentrations (from 0.1 to 100 µM) for 72 h, was evaluated by SRB assay. Logarithmic scale was used for data presentation

Then, the effect of darifenacin in primary culture cells was evaluated, using the SRB assay. Results (Fig. 3) demonstrated that darifenacin significantly inhibited the growth of PDAC-3 primary cells (GI_50_ concentration of 30 µM) following a 72 h incubation period. This data reinforces our previous results suggesting that darifenacin interferes with PDAC cellular growth at concentrations within the µM range.

### Darifenacin does not exert major cytotoxic effects in human non-tumorigenic cells

**Fig. 4:**
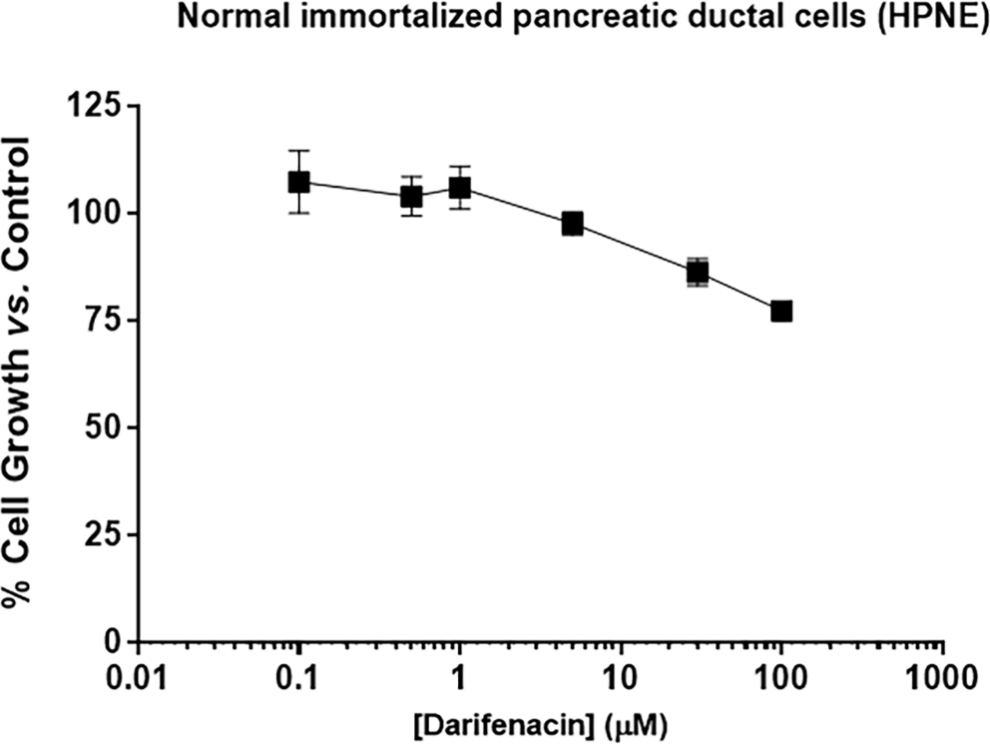
Dose-response curve of HPNE cells following treatment with darifenacin. The percentage (%) of growth of the cells treated with darifenacin, at various concentrations (from 0.1 to 100 µM) for 72 h, was evaluated by SRB assay. Logarithmic scale was used for data presentation

The normal immortalized pancreatic ductal cells (HPNE) were used to determine the cytotoxic effect of darifenacin, using the SRB assay. Our data (Fig. 4) revealed that darifenacin did not significantly affect the % of cell growth of HPNE cells at concentrations below 50 µM.

Moreover, we observed that darifenacin at 8 µM and 10 µM did not cause a significant effect on the growth of the non-tumorigenic epithelial cell line MCF-10 A, determined by the SRB assay (Supplementary Fig. 1).

### Chemosensitizing effect of darifenacin in combination with gemcitabine or gemcitabine plus paclitaxel

**Fig. 5:**
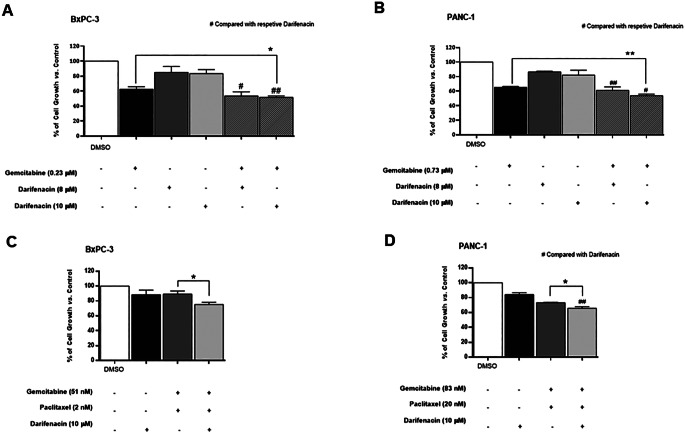
Effect of the combination treatment consisting of darifenacin with gemcitabine or with gemcitabine plus paclitaxel in BxPC-3 (**A**, **C**) and PANC-1 (**B**, **D**) pancreatic cancer cell lines, as determined by the SRB assay. Incubation for 48 h with Control (vehicle, DMSO), darifenacin (at 8 and 10 µM), gemcitabine at its GI_50_ concentrations (0.23 µM in BxPC-3 and 0.73 µM in PANC-1) and at its GI_25_ concentrations (51 nM in BxPC-3 and 83 nM in PANC-1) and paclitaxel at its GI_25_ concentrations (2 nM in BxPC-3 and 20 nM in PANC-1). Results correspond to the mean ± S.E.M. of at least three independent experiments. *,# *p* ≤ 0.05 and **,## *p* ≤ 0.01 of Control vs. Treatments

Our previous data showed that pentoxifylline reverts gemcitabine resistance induced by the human recombinant protein for CHI3L1 (rhCHI3L1) in PDAC cells [9]. We also observed that the combination of gemcitabine with different concentrations of pentoxifylline (a known CHI3L1 inhibitor) significantly reduced BxPC-3 cell growth (Supplementary Fig. 2), suggesting that CHI3L1 inhibitors may overcome gemcitabine resistance in PDAC cells. Hence, darifenacin at two concentrations (8 µM and 10 µM) was tested in combination with the GI_50_ concentrations of gemcitabine (concentrations previously described [9]), and cell growth measured by SRB assay. As shown in Fig. 5A and B, the combination of gemcitabine with darifenacin at the highest concentration tested (10 µM) significantly reduced the % of cell growth of BxPC-3 and PANC-1 cell lines, when compared with the individual treatments.

Since combination treatment of gemcitabine plus paclitaxel is also considered a treatment option for PDAC patients [42], we further evaluated the possible chemosensitizing effect of darifenacin to this drug combination (gemcitabine plus paclitaxel). For that, darifenacin at the highest concentration tested (10 µM) was combined with the GI_25_ concentrations of gemcitabine (51 nM in BxPC-3 and 83 nM in PANC-1) [9] and the GI_25_ concentrations of paclitaxel (2 nM in BxPC-3 and 20 nM in PANC-1; extrapolated by the dose-response curve; GI_50_ is presented in Supplementary Table 1) in the BxPC-3 and PANC-1, followed by the SRB assay. Our results demonstrated that darifenacin (at 10 µM) in combination with gemcitabine and paclitaxel significantly reduced the % of cell growth in both pancreatic cell lines, when compared with the treatment consisting of gemcitabine and paclitaxel (Fig. 5C and D).

### Involvement of CHI3L1 and its downstream Akt protein in the chemosensitizing effect of darifenacin

**Fig. 6:**
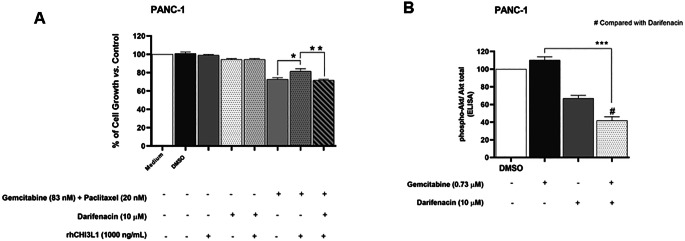
Mechanism of action behind the chemosensitizing effect of darifenacin. **(A)** Effect of 1000 ng/ml of rhCHI3L1 in the presence or absence of: (**a**) darifenacin at 10 µM; (**b**) gemcitabine at 83 nM plus paclitaxel at 20 nM; or (**c**) darifenacin at 10 µM with gemcitabine at 83 nM plus paclitaxel at 20 nM. Vehicle (at the highest concentration tested) and medium alone were used as negative controls. Results are the mean ± S.E.M of at least three independent experiments. *p < 0.05; **p < 0.01. **(B)** Akt phosphorylation following treatment with (i) gemcitabine at 0.73 µM; (ii) darifenacin at 10 µM; or (iii) gemcitabine at 0.73 µM plus darifenacin at 10 µM. Vehicle was used as negative control. Results are the mean ± S.E.M of at least three independent experiments. *p < 0.05; **p < 0.01

To confirm the involvement of CHI3L1 on PDAC cellular resistance to gemcitabine plus paclitaxel, and to assess the influence of darifenacin on CHI3L1-induced PDAC cellular resistance to treatment, the rhCHI3L1 (mimicking the presence of this protein) was used and SRB assay was performed. We observed that rhCHI3L1 induced resistance to gemcitabine plus paclitaxel treatment in BxPC-3 cells (Supplementary Fig. 3). Our results (Fig. 6A) also demonstrated that rhCHI3L1 significantly increased PANC-1 cellular resistance to the combination treatment of gemcitabine plus paclitaxel, which was statistically significantly abrogated by darifenacin treatment.

Since CHI3L1 is known to act through activation of the Akt signaling pathway [43], the potential effect of darifenacin on Akt protein activation was further analyzed in PANC-1 cells by ELISA. Our data (Fig. 6B) revealed that darifenacin at 10 µM reduced Akt phosphorylation, contrarily to what was observed for gemcitabine alone (at 0.73 µM). Remarkably, gemcitabine in combination with darifenacin statistically significantly reduced Akt protein activation, when compared to each individual treatment, suggesting that the chemosensitizing effect of darifenacin may be related to the inhibition of Akt pathway.

### Clinical relevance of CHRM3 expression in human PDAC tissue samples treated with gemcitabine

**Fig. 7:**
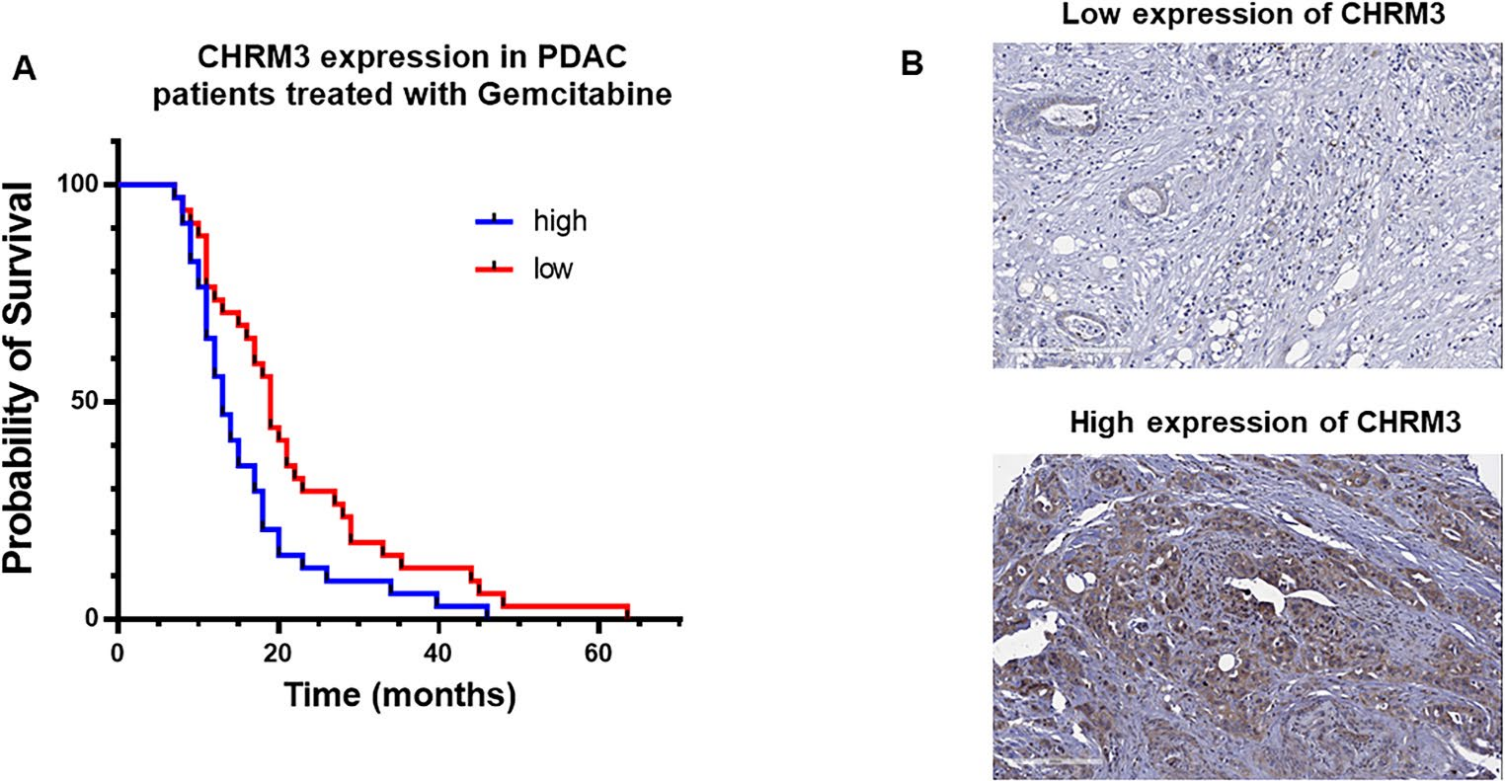
Clinical relevance of CHRM3 expression in human PDAC. **(A)** Survival curves of PDAC patients treated with gemcitabine with high or low CHRM3 (Cholinergic Receptor Muscarinic 3) expression (*n* = 68, * *p* < 0.017); **(B)** Representative images showing differential expression (high vs. low) of CHRM3 in PDAC specimens. Original magnification 10x

Darifenacin is a well-known CHRM3 antagonist that is clinically approved for the treatment of overactive bladder and urinary incontinence [44]. Given the previously presented antitumoral and chemosentizing effect of darifenacin, we proceeded to investigate the association between CHRM3 expression and PDAC therapeutic response to gemcitabine. For that, we performed IHC of paraffin-embedded tissues from surgical resections of a patients’ cohort (Supplementary Table 2). Remarkably, high expression of CRHM3 in PDAC patient tissues was significantly (* *p* < 0.05) related with reduced therapeutic response to gemcitabine (Fig. 7).

## Discussion

CHI3L1 is known to be overexpressed in patients with PDAC and it has been associated with a poor prognosis [10, 11]. Our previous work demonstrated that CHI3L1 is implicated in PDAC cellular resistance to gemcitabine, identifying CHI3L1 as a potential therapeutic target for the treatment of this type of cancer [9]. Importantly, a recent study verified that human monoclonal CHI3L1 neutralizing antibodies (nAbs) inhibited the tumor growth as well as mitigated fibrosis, angiogenesis and restored immunostimulatory functions in a pancreatic cancer orthotopic mice model [45]. Therefore, the identification of CHI3L1 inhibitors could be of great interest to provide new therapeutic options, as well as to overcome drug resistance in PDAC.

The 2.20 Å resolution structure of homo sapiens CHI3L1 (pdb code: 1NWU) was selected to perform a virtual screening on a database of known compounds to search of potential new CHI3L1 inhibitors [35]. The docking study (Autodock Vina) allowed the evaluation of possible poses within the binding pocket of CHI3L1 and the prediction of the binding strength of thousands of known compounds. Interestingly, a study of the crystal structure with a long chitin fragment demonstrated that TRP 99, ASN 100, TYR 141, ARG 263 and TRP 352 participate in ligand recognition [20]. Some of the known ligands used in our study were able to interact with those CHI3L1 residues (represented in green, in Supplementary Fig. 4). Moreover, it is possible to verify that chitotetraose and chitohexaose interact with almost all the residues already described since they are chitin-like oligosaccharides. Through molecular docking of 11,741 molecules retrieved from DrugBank, we identified darifenacin as a compound with the potential ability to strongly bind to CHI3L1. Darifenacin showed a higher affinity towards CHI3L1 when compared to the ligand prednisolone (the positive control used as the threshold for this virtual screening) and other positive controls (Table 2). Moreover, in accordance with previous literature, this drug interacted with the residues TRP 99, ASN 100 and TRP 352 that are involving the chitin fragment [20], as also observed for the positive controls (Supplementary Fig. 4). Interestingly, most of the residues that intervene in the binding of positive controls to CHI3L1 were not involved in the binding of darifenacin. Moreover, this drug was able to establish an interaction with a different residue, LEU 140 (Supplementary Fig. 4), which may strengthen the binding of darifenacin to the target. Therefore, and since darifenacin showed a lower score value compared with the positive controls, it was selected for further in vitro experiments.

Darifenacin is a clinically approved drug for the treatment of overactive bladder and urinary incontinence [46]. Importantly, the antitumor activity of darifenacin has already been described for other cancer types, including colorectal [47], gastric [48, 49] and lung [34, 50] cancers. For instance, darifenacin blocks the muscarinic acetylcholine receptor 3 in colorectal cancer cells, leading to a decrease in the p38, Erk and Akt signaling pathways, as well as the disruption of MMP-1 mRNA expression. In this work, the inhibition of tumor growth was also observed using human colon adenocarcinoma xenograft models treated with darifenacin [46]. Other studies also reported that darifenacin inhibited the invasion of gastric cancer cells and the expression of epithelial-mesenchymal transition (EMT) markers that was promoted by acetylcholine [47]. Darifenacin also markedly inhibited the formation of gastric tumors in vivo [49]. In addition, darifenacin inhibited the growth of small cell lung cancer xenografts and decreased MAPK phosphorylation in vivo [50]. However, the potential antitumor effect of darifenacin in PDAC and the advantage of use this drug in combination with chemotherapeutic drugs was unknown.

Our data demonstrated that darifenacin decreased the growth of PDAC cells. Remarkably, darifenacin, a non-anticancer drug, hindered the growth of two immortalized PDAC cell lines at a concentration below 30 µM. Furthermore, darifenacin had little or no effect against non-tumorigenic pancreatic cells, suggesting that this drug, while influencing PDAC cells growth, may have minor toxic effects against non-tumor cells. Interestingly, PANC-1 cells, which have mutated *KRAS* (Kirsten rat sarcoma virus) and are associated with a more aggressive phenotype [51], demonstrated higher sensitivity to darifenacin (GI_50_ of 13.6 µM) than the less resistant BxPC-3 cells (wild-type for *KRAS*; GI_50_ of 26 µM). Importantly, the cytotoxic effect of darifenacin was further validated in PDAC-3 primary culture cells, which are more representative of the human onset heterogeneity.

In recent years, combination therapies have caught the attention of the cancer research community as a strategy to simultaneously target different targets in cancer cells, allowing in some cases to overcome drug resistance [52]. In this work, we demonstrated that darifenacin may sensitize PDAC cell lines to gemcitabine or to gemcitabine plus paclitaxel treatments, highlighting the chemosensitizing effect of this drug in PDAC. In addition to the high levels of CHI3L1 expression already reported in PDAC tumors [10, 11], this study evaluated the presence of muscarinic acetylcholine receptors (mAChRs) in PDAC tumors and its association with drug treatment. The impact of the cholinergic signaling via mAChRs in the progression of cancer has already been proposed [53–55], supporting the idea that inhibitors of the mAChRs could be clinically useful for the treatment of certain types of cancer. Notably, darifenacin is known to be an antagonist of the cholinergic receptor muscarinic 3 (CHRM3) [56]. For instance, high expression levels of CHRM3 have been found to predict poor prognosis in patients with pancreatic cancer, suggesting CHRM3 as a potential prognostic marker [57]. Consistent with these results, our data revealed a correlation between high expression levels of CHRM3 and lower survival rates in a group of 68 PDAC patients treated with gemcitabine. Therefore, our study suggests that increased expression of this receptor might be linked to a reduced response to chemotherapy, further emphasizing the potential usefulness of darifenacin in PDAC therapy.

Collectively, our preclinical in vitro investigations have highlighted the potential of repurposing darifenacin in combination with gemcitabine or with gemcitabine plus paclitaxel for treating pancreatic cancer. Darifenacin is an approved drug with an established safety profile, having documented clinical efficacy and toxicological information. To further validate our preclinical data and lay the groundwork for a potential clinical trial, additional preclinical studies utilizing more complex biological models, such as xenografted mice models are needed. Nevertheless, as far as we know, this in vitro work is the first to explore the prospect of repurposing darifenacin for pancreatic cancer treatment.

## Electronic supplementary material

Below is the link to the electronic supplementary material.

Supplementary Material 1

## Data Availability

No datasets were generated or analysed during the current study.

## References

[1] Siegel RL, Miller KD, Wagle NS, Jemal A (2023) Cancer statistics, 2023. CA: a cancer journal for clinicians 73. 117–48. 10.3322/caac.21763 10.3322/caac.21763 36633525

[2] Kunovsky L, Tesarikova P, Kala Z, Kroupa R, Kysela P, Dolina J, Trna J (2018) The use of biomarkers in Early Diagnostics of Pancreatic Cancer. Can J Gastroenterol Hepatol 2018:5389820–5389820. 10.1155/2018/5389820 30186820 10.1155/2018/5389820 PMC6112218

[3] Halbrook CJ, Lyssiotis CA, Pasca di Magliano M, Maitra A (2023) Pancreatic cancer: advances and challenges. Cell 186(8):1729–1754. 10.1016/j.cell.2023.02.014 37059070 10.1016/j.cell.2023.02.014 PMC10182830

[4] Sarantis P, Koustas E, Papadimitropoulou A, Papavassiliou AG, Karamouzis MV (2020) Pancreatic ductal adenocarcinoma: treatment hurdles, tumor microenvironment and immunotherapy. World J Gastrointest Oncol 12(2):173–181. 10.4251/wjgo.v12.i2.173 32104548 10.4251/wjgo.v12.i2.173 PMC7031151

[5] Capula M, Peran M, Xu G, Donati V, Yee D, Gregori A, Assaraf YG, Giovannetti E, Deng D (2022) Role of drug catabolism, modulation of oncogenic signaling and tumor microenvironment in microbe-mediated pancreatic cancer chemoresistance. Drug Resist Updat 64:100864. 10.1016/j.drup.2022.100864 36115181 10.1016/j.drup.2022.100864

[6] Rebelo R, Xavier CPR, Giovannetti E, Vasconcelos MH (2023) Fibroblasts in pancreatic cancer: molecular and clinical perspectives. Trends Mol Med. 10.1016/j.molmed.2023.03.002 37100646 10.1016/j.molmed.2023.03.002

[7] Adamska A, Domenichini A, Falasca M (2017) Pancreatic ductal adenocarcinoma: current and evolving therapies. Int J Mol Sci 18(7):1338. 10.3390/ijms18071338 28640192 10.3390/ijms18071338 PMC5535831

[8] Vogl UM, Andalibi H, Klaus A, Vormittag L, Schima W, Heinrich B, Kafka A, Winkler T, Öhler L (2019) Nab-paclitaxel and gemcitabine or FOLFIRINOX as first-line treatment in patients with unresectable adenocarcinoma of the pancreas: does sequence matter? BMC Cancer 19(1):28–28. 10.1186/s12885-018-5240-6 30621630 10.1186/s12885-018-5240-6 PMC6325849

[9] Xavier CPR, Castro I, Caires HR, Ferreira D, Cavadas B, Pereira L, Santos LL, Oliveira MJ, Vasconcelos MH (2021) Chitinase 3-like-1 and fibronectin in the cargo of extracellular vesicles shed by human macrophages influence pancreatic cancer cellular response to gemcitabine. Cancer Lett 501:210–223. 10.1016/j.canlet.2020.11.013 33212158 10.1016/j.canlet.2020.11.013

[10] Chen IM, Johansen AZ, Dehlendorff C, Jensen BV, Bojesen SE, Pfeiffer P, Bjerregaard JK, Nielsen SE, Andersen F, Hollander NH, Yilmaz MK, Rasmussen LS, Johansen JS (2020) Prognostic value of combined detection of serum IL6, YKL-40, and C-reactive protein in patients with unresectable pancreatic Cancer. Cancer Epidemiol Biomarkers Prev 29(1):176–184. 10.1158/1055-9965.EPI-19-0672 31685562 10.1158/1055-9965.EPI-19-0672

[11] Chen HT, Zheng JM, Zhang YZ, Yang M, Wang YL, Man XH, Chen Y, Cai QC, Li ZS (2017) Overexpression of YKL-40 predicts poor prognosis in patients undergoing curative resection of pancreatic Cancer. Pancreas 46(3):323–334. 10.1097/MPA.0000000000000751 28099248 10.1097/MPA.0000000000000751

[12] Yu JE, Yeo IJ, Han SB, Yun J, Kim B, Yong YJ, Lim YS, Kim TH, Son DJ, Hong JT (2024) Significance of chitinase-3-like protein 1 in the pathogenesis of inflammatory diseases and cancer. Exp Mol Med 56(1):1–18. 10.1038/s12276-023-01131-9 38177294 10.1038/s12276-023-01131-9 PMC10834487

[13] Chang MC, Chen CT, Chiang PF, Chiang YC (2024) The role of Chitinase-3-like Protein-1 (YKL40) in the therapy of Cancer and other chronic-inflammation-related diseases. Pharmaceuticals (Basel) 17(3). 10.3390/ph17030307 10.3390/ph17030307 PMC10976000 38543093

[14] Zhao T, Su Z, Li Y, Zhang X, You Q (2020) Chitinase-3 like-protein-1 function and its role in diseases. Signal Transduct Target Ther 5(1):201. 10.1038/s41392-020-00303-7 32929074 10.1038/s41392-020-00303-7 PMC7490424

[15] Di Rosa M, Distefano G, Zorena K, Malaguarnera L (2016) Chitinases and immunity: ancestral molecules with new functions. Immunobiology 221(3):399–411. 10.1016/j.imbio.2015.11.014 26686909 10.1016/j.imbio.2015.11.014

[16] Hakala BE, White C, Recklies AD (1993) Human cartilage gp-39, a major secretory product of articular chondrocytes and synovial cells, is a mammalian member of a chitinase protein family. J Biol Chem 268(34):25803–25810 8245017

[17] Renkema GH, Boot RG, Au FL, Donker-Koopman WE, Strijland A, Muijsers AO, Hrebicek M, Aerts JM (1998) Chitotriosidase, a chitinase, and the 39-kDa human cartilage glycoprotein, a chitin-binding lectin, are homologues of family 18 glycosyl hydrolases secreted by human macrophages. Eur J Biochem 251(1–2):504–509. 10.1046/j.1432-1327.1998.2510504.x 9492324 10.1046/j.1432-1327.1998.2510504.x

[18] Fusetti F, Pijning T, Kalk KH, Bos E, Dijkstra BW (2003) Crystal structure of human cartilage gp39 (HC-gp39) in complex with chitotetraose. https://www.rcsb.org/structure/1NWU 10.1074/jbc.M303137200 12851408

[19] Houston DR, Recklies AD, Krupa JC, van Aalten DM (2003) Structure and ligand-induced conformational change of the 39-kDa glycoprotein from human articular chondrocytes. J Biol Chem 278(32):30206–30212. 10.1074/jbc.M303371200 12775711 10.1074/jbc.M303371200

[20] Fusetti F, Pijning T, Kalk KH, Bos E, Dijkstra BW (2003) Crystal structure and carbohydrate-binding properties of the human cartilage glycoprotein-39. J Biol Chem 278(39):37753–37760. 10.1074/jbc.M303137200 12851408 10.1074/jbc.M303137200

[21] Rebelo R, Polónia B, Santos LL, Vasconcelos MH, Xavier CPR (2021) Drug Repurposing opportunities in Pancreatic Ductal Adenocarcinoma. Pharmaceuticals (Basel) 14(3):280 33804613 10.3390/ph14030280 PMC8003696

[22] Forli S, Huey R, Pique ME, Sanner MF, Goodsell DS, Olson AJ (2016) Computational protein-ligand docking and virtual drug screening with the AutoDock suite. Nat Protoc 11(5):905–919. 10.1038/nprot.2016.051 27077332 10.1038/nprot.2016.051 PMC4868550

[23] Wishart DS, Feunang YD, Guo AC, Lo EJ, Marcu A, Grant JR, Sajed T, Johnson D, Li C, Sayeeda Z, Assempour N, Iynkkaran I, Liu Y, Maciejewski A, Gale N, Wilson A, Chin L, Cummings R, Le D, Pon A, Knox C, Wilson M (2018) DrugBank 5.0: a major update to the DrugBank database for 2018. Nucleic Acids Res 46(D1):D1074–d1082. 10.1093/nar/gkx1037 29126136 10.1093/nar/gkx1037 PMC5753335

[24] Trott O, Olson AJ (2010) AutoDock Vina: improving the speed and accuracy of docking with a new scoring function, efficient optimization, and multithreading. J Comput Chem 31(2):455–461. 10.1002/jcc.21334 19499576 10.1002/jcc.21334 PMC3041641

[25] Seeliger D, de Groot BL (2010) Ligand docking and binding site analysis with PyMOL and Autodock/Vina. J Comput Aided Mol Des 24(5):417–422. 10.1007/s10822-010-9352-6 20401516 10.1007/s10822-010-9352-6 PMC2881210

[26] Lill MA, Danielson ML (2011) Computer-aided drug design platform using PyMOL. J Comput Aided Mol Des 25(1):13–19. 10.1007/s10822-010-9395-8 21053052 10.1007/s10822-010-9395-8

[27] Branco H, Oliveira J, Antunes C, Santos LL, Vasconcelos MH, Xavier CPR (2022) Pirfenidone sensitizes NCI-H460 Non-small Cell Lung Cancer cells to Paclitaxel and to a combination of Paclitaxel with Carboplatin. Int J Mol Sci 23(7). 10.3390/ijms23073631 10.3390/ijms23073631 PMC8998757 35408988

[28] Rovithi M, Avan A, Funel N, Leon LG, Gomez VE, Wurdinger T, Griffioen AW, Verheul HM, Giovannetti E (2017) Development of bioluminescent chick chorioallantoic membrane (CAM) models for primary pancreatic cancer cells: a platform for drug testing. Sci Rep 7:44686. 10.1038/srep44686 28304379 10.1038/srep44686 PMC5356332

[29] Silva BR, Rebelo R, Rodrigues JM, Xavier CPR, Vasconcelos MH, Queiroz MRP (2021) Synthesis of Novel Methyl 3-(hetero)arylthieno[3,2-b]pyridine-2-carboxylates and antitumor activity evaluation: studies in Vitro and in Ovo grafts of Chick Chorioallantoic membrane (CAM) with a Triple negative breast Cancer Cell line. Molecules 26(6). 10.3390/molecules26061594 10.3390/molecules26061594 PMC7999514 33805741

[30] Vichai V, Kirtikara K (2006) Sulforhodamine B colorimetric assay for cytotoxicity screening. Nat Protoc 1(3):1112–1116. 10.1038/nprot.2006.179 17406391 10.1038/nprot.2006.179

[31] Massihnia D, Avan A, Funel N, Maftouh M, van Krieken A, Granchi C, Raktoe R, Boggi U, Aicher B, Minutolo F, Russo A, Leon LG, Peters GJ, Giovannetti E (2017) Phospho-akt overexpression is prognostic and can be used to tailor the synergistic interaction of akt inhibitors with gemcitabine in pancreatic cancer. J Hematol Oncol 10(1):9. 10.1186/s13045-016-0371-1 28061880 10.1186/s13045-016-0371-1 PMC5219723

[32] Ali A, Jamieson NB, Khan IN, Chang D, Giovannetti E, Funel N, Frampton AE, Morton J, Sansom O, Evans TRJ, Duthie F, McKay CJ, Samra J, Gill AJ, Biankin A, Oien KA (2022) Prognostic implications of microRNA-21 overexpression in pancreatic ductal adenocarcinoma: an international multicenter study of 686 patients. Am J Cancer Res 12(12):5668–5683 36628279 PMC9827095

[33] Le Large TYS, El Hassouni B, Funel N, Kok B, Piersma SR, Pham TV, Olive KP, Kazemier G, van Laarhoven HWM, Jimenez CR, Bijlsma MF, Giovannetti E (2019) Proteomic analysis of gemcitabine-resistant pancreatic cancer cells reveals that microtubule-associated protein 2 upregulation associates with taxane treatment. Ther Adv Med Oncol 11:1758835919841233. 10.1177/1758835919841233 31205498 10.1177/1758835919841233 PMC6535709

[34] Song P, Sekhon HS, Lu A, Arredondo J, Sauer D, Gravett C, Mark GP, Grando SA, Spindel ER (2007) M3 muscarinic receptor antagonists inhibit small cell lung carcinoma growth and mitogen-activated protein kinase phosphorylation induced by acetylcholine secretion. Cancer Res 67(8):3936–3944. 10.1158/0008-5472.CAN-06-2484 17440109 10.1158/0008-5472.CAN-06-2484

[35] Crystal structure of human cartilage (2003) gp39 (HC-gp39) in complex with chitotetraose https://www.rcsb.org/structure/1NWU

[36] Kognole AA, Payne CM (2017) Inhibition of mammalian glycoprotein YKL-40: IDENTIFICATION OF THE PHYSIOLOGICAL LIGAND. J Biol Chem 292(7):2624–2636. 10.1074/jbc.M116.764985 28053085 10.1074/jbc.M116.764985 PMC5314161

[37] Lee IA, Kamba A, Low D, Mizoguchi E (2014) Novel methylxanthine derivative-mediated anti-inflammatory effects in inflammatory bowel disease. World J Gastroenterol 20(5):1127–1138. 10.3748/wjg.v20.i5.1127 24574789 10.3748/wjg.v20.i5.1127 PMC3921497

[38] Zadi Heydarabad M, Baharaghdam S, Azimi A, Mohammadi H, Eivazi Ziaei J, Yazdanpanah B, Zak MS, Farahani ME, Dohrabpour A, Partash N, Talebi M (2019) The role of tumor suppressor of resveratrol and prednisolone by downregulation of YKL-40 expression in CCRF-CEM cell line. J Cell Biochem 120(3):3773–3779. 10.1002/jcb.27659 30426549 10.1002/jcb.27659

[39] Zhang W, Murao K, Zhang X, Matsumoto K, Diah S, Okada M, Miyake K, Kawai N, Fei Z, Tamiya T (2010) Resveratrol represses YKL-40 expression in human glioma U87 cells. BMC Cancer 10:593. 10.1186/1471-2407-10-593 21029458 10.1186/1471-2407-10-593 PMC2988030

[40] Rao FV, Andersen OA, Vora KA, Demartino JA, van Aalten DM (2005) Methylxanthine drugs are chitinase inhibitors: investigation of inhibition and binding modes. Chem Biol 12(9):973–980. 10.1016/j.chembiol.2005.07.009 16183021 10.1016/j.chembiol.2005.07.009

[41] Yamada S, Ito Y, Nishijima S, Kadekawa K, Sugaya K (2018) Basic and clinical aspects of antimuscarinic agents used to treat overactive bladder. Pharmacol Ther 189:130–148. 10.1016/j.pharmthera.2018.04.010 29709423 10.1016/j.pharmthera.2018.04.010

[42] Von Hoff DD, Ervin T, Arena FP, Chiorean EG, Infante J, Moore M, Seay T, Tjulandin SA, Ma WW, Saleh MN, Harris M, Reni M, Dowden S, Laheru D, Bahary N, Ramanathan RK, Tabernero J, Hidalgo M, Goldstein D, Van Cutsem E, Wei X, Iglesias J, Renschler MF (2013) Increased survival in pancreatic cancer with nab-paclitaxel plus gemcitabine. N Engl J Med 369(18):1691–1703. 10.1056/NEJMoa1304369 24131140 10.1056/NEJMoa1304369 PMC4631139

[43] Geng B, Pan J, Zhao T, Ji J, Zhang C, Che Y, Yang J, Shi H, Li J, Zhou H, Mu X, Xu C, Wang C, Xu Y, Liu Z, Wen H, You Q (2018) Chitinase 3-like 1-CD44 interaction promotes metastasis and epithelial-to-mesenchymal transition through beta-catenin/Erk/Akt signaling in gastric cancer. J Exp Clin Cancer Res 37(1):208. 10.1186/s13046-018-0876-2 30165890 10.1186/s13046-018-0876-2 PMC6117920

[44] Yamada S, Ito Y, Nishijima S, Kadekawa K, Sugaya K (2018) Basic and clinical aspects of antimuscarinic agents used to treat overactive bladder. Pharmacol Ther 189:130–148. 10.1016/j.pharmthera.2018.04.010 29709423 10.1016/j.pharmthera.2018.04.010

[45] Su PC, Chen CY, Yu MH, Kuo IY, Yang PS, Hsu CH, Hou YC, Hsieh HT, Chang CP, Shan YS, Wang YC (2024) Fully human chitinase-3 like-1 monoclonal antibody inhibits tumor growth, fibrosis, angiogenesis, and immune cell remodeling in lung, pancreatic, and colorectal cancers. Biomed Pharmacother 176:116825. 10.1016/j.biopha.2024.116825 38820971 10.1016/j.biopha.2024.116825

[46] Zinner N, Kobashi KC, Ebinger U, Viegas A, Egermark M, Quebe-Fehling E, Koochaki P (2008) Darifenacin treatment for overactive bladder in patients who expressed dissatisfaction with prior extended-release antimuscarinic therapy. Int J Clin Pract 62(11):1664–1674. 10.1111/j.1742-1241.2008.01893.x 18811599 10.1111/j.1742-1241.2008.01893.x PMC2680263

[47] Hering NA, Liu V, Kim R, Weixler B, Droeser RA, Arndt M, Pozios I, Beyer K, Kreis ME, Seeliger H (2021) Blockage of Cholinergic Signaling via Muscarinic Acetylcholine Receptor 3 inhibits Tumor Growth in Human Colorectal Adenocarcinoma. Cancers 13(13):3220. 10.3390/cancers13133220 34203220 10.3390/cancers13133220 PMC8267754

[48] Yang T, He W, Cui F, Xia J, Zhou R, Wu Z, Zhao Y, Shi M (2016) MACC1 mediates acetylcholine-induced invasion and migration by human gastric cancer cells. Oncotarget 7(14):18085–18094. 10.18632/oncotarget.7634 26919111 10.18632/oncotarget.7634 PMC4951273

[49] Yu H, Xia H, Tang Q, Xu H, Wei G, Chen Y, Dai X, Gong Q, Bi F (2017) Acetylcholine acts through M3 muscarinic receptor to activate the EGFR signaling and promotes gastric cancer cell proliferation. Sci Rep 7:40802. 10.1038/srep40802 28102288 10.1038/srep40802 PMC5244394

[50] Song P, Sekhon HS, Fu XW, Maier M, Jia Y, Duan J, Proskosil BJ, Gravett C, Lindstrom J, Mark GP, Saha S, Spindel ER (2008) Activated cholinergic signaling provides a target in squamous cell lung carcinoma. Cancer Res 68(12):4693–4700. 10.1158/0008-5472.CAN-08-0183 18559515 10.1158/0008-5472.CAN-08-0183 PMC2865551

[51] Sipos B, Moser S, Kalthoff H, Torok V, Lohr M, Kloppel G (2003) A comprehensive characterization of pancreatic ductal carcinoma cell lines: towards the establishment of an in vitro research platform. Virchows Arch 442(5):444–452. 10.1007/s00428-003-0784-4 12692724 10.1007/s00428-003-0784-4

[52] Bhatia K, Bhumika, Das A (2020) Combinatorial drug therapy in cancer - new insights. Life Sci 258:118134. 10.1016/j.lfs.2020.118134 32717272 10.1016/j.lfs.2020.118134

[53] Matera C, Tata AM (2014) Pharmacological approaches to targeting muscarinic acetylcholine receptors. Recent Pat CNS Drug Discov 9(2):85–100. 10.2174/1574889809666141120131238 25413004 10.2174/1574889809666141120131238

[54] Schledwitz A, Sundel MH, Alizadeh M, Hu S, Xie G, Raufman JP (2021) Differential actions of muscarinic receptor subtypes in gastric, pancreatic, and Colon cancer. Int J Mol Sci 22(23). 10.3390/ijms222313153 10.3390/ijms222313153 PMC8658119 34884958

[55] Kruse AC, Kobilka BK, Gautam D, Sexton PM, Christopoulos A, Wess J (2014) Muscarinic acetylcholine receptors: novel opportunities for drug development. Nat Rev Drug Discov 13(7):549–560. 10.1038/nrd4295 24903776 10.1038/nrd4295 PMC5818261

[56] Chapple CR (2004) Darifenacin: a novel M3 muscarinic selective receptor antagonist for the treatment of overactive bladder. Expert Opin Investig Drugs 13(11):1493–1500. 10.1517/13543784.13.11.1493 15500396 10.1517/13543784.13.11.1493

[57] Zhang L, Xiu D, Zhan J, He X, Guo L, Wang J, Tao M, Fu W, Zhang H (2016) High expression of muscarinic acetylcholine receptor 3 predicts poor prognosis in patients with pancreatic ductal adenocarcinoma. Onco Targets Ther 9:6719–6726. 10.2147/OTT.S111382 27826198 10.2147/OTT.S111382 PMC5096762

