# Supplementary material for: Darifenacin: a promising chitinase 3-like 1 inhibitor to tackle drug resistance in pancreatic ductal adenocarcinoma
Source: Cancer Chemother Pharmacol. 2024 Sep 3;94(4):585–97. doi: 10.1007/s00280-024-04712-1 (PMC11438711; doi:10.1007/s00280-024-04712-1)
Supplement: Supplementary file 1 — Supplementary Material 1 [file 280_2024_4712_MOESM1_ESM.docx]

*Supplementary Data*

Darifenacin: A promising Chitinase 3-like 1 inhibitor to tackle drug resistance in pancreatic ductal adenocarcinoma

Sofia M. Sousa ^1,2,3,#^, Helena Branco ^1,2,#^, Amir Avan ^4,5^, Andreia Palmeira ^3,6^, Luca Morelli ^7^, Lúcio L.

Santos ^8,9^, Elisa Giovannetti ^10,11^, M. Helena Vasconcelos ^1,2,12,^* and Cristina P. R. Xavier ^1,2,13,14^*

*^1^i3S – Instituto de Investigação e Inovação em Saúde, Universidade do Porto, Rua Alfredo Allen 208, Porto, 4200-135, Portugal;
^2^ Cancer Drug Resistance Group, IPATIMUP - Institute of Molecular Pathology and Immunology of the University of Porto, Rua Alfredo Allen 208, Porto, 4200-135, Portugal;
^3^ LQOF – Laboratory of Organic and Pharmaceutical Chemistry, Department of Chemical Sciences, Faculty of Pharmacy, University of Porto, Rua de Jorge Viterbo Ferreira 228, Porto, 4050-313, Portugal;
^4^ Metabolic Syndrome Research Center, Mashhad University of Medical Sciences, Mashhad, 91886-17871, Iran;
^5^ Medical Genetics Research Center, Faculty of Medicine, Mashhad University of Medical Sciences, Mashhad, 91886-17871, Iran;
^6^ CIIMAR – Interdisciplinary Centre of Marine and Environmental Research, Terminal de Cruzeiros do Porto de Leixões, Matosinhos, 4450-208, Portugal;
^7^ General Surgery Unit, Department of Translational Research and New Technologies in Medicine and Surgery, University of Pisa, Pisa, 56100, Italy;
^8^ Experimental Pathology and Therapeutics Research Group and Surgical Oncology Department, IPO—Instituto Português de Oncologia, Rua Dr. António Bernardino de Almeida 865, Porto, 4200-072, Portugal;
^9^ ICBAS-UP—School of Medicine and Biomedical Sciences, University of Porto, Rua de Jorge Viterbo Ferreira 228, Porto, 4050-313, Portugal;
^10^ Department of Medical Oncology, Cancer Center Amsterdam, Amsterdam UMC, Vrije Universiteit, HV Amsterdam, 1081, The Netherlands;
^11^ Cancer Pharmacology Lab, Fondazione Pisana per La Scienza, San Giuliano, 56017, Italy;
^12^ Department of Biological Sciences, FFUP – Faculty of Pharmacy, University of Porto, Rua de Jorge Viterbo Ferreira 228, Porto, 4050-313, Portugal;
^13^ UCIBIO - Applied Molecular Biosciences Unit, Toxicologic Pathology Research Laboratory, University Institute of Health Sciences (1H-TOXRUN, IUCS-CESPU), 4585-116 Gandra, Portugal;
^14^ Associate Laboratory i4HB - Institute for Health and Bioeconomy, University Institute of Health Sciences - CESPU, 4585-116 Gandra, Portugal;*

*# These authors equally contributed to this work.*

**Correspondence to:** Prof. Doctor M. Helena Vasconcelos, *i3S – Instituto de Investigação e Inovação em Saúde, Universidade do Porto,* Rua Alfredo Allen 208, Porto, 4200-135, Portugal; E-mail: hvasconcelos@ipatimup.pt; ORCID: 0000-0002-7801-4643. Prof. Doctor Cristina P.R. Xavier, *i3S – Instituto de Investigação e Inovação em Saúde, Universidade do Porto,* Rua Alfredo Allen 208, Porto, 4200-135, Portugal and *1H-TOXRUN, IUCS-CESPU, 4585-116 Gandra, Portugal.* E-mail: cristina.xavier@iucs.cespu.pt; ORCID: 0000-0002-4613-1917.

***Supplementary Figures***

**
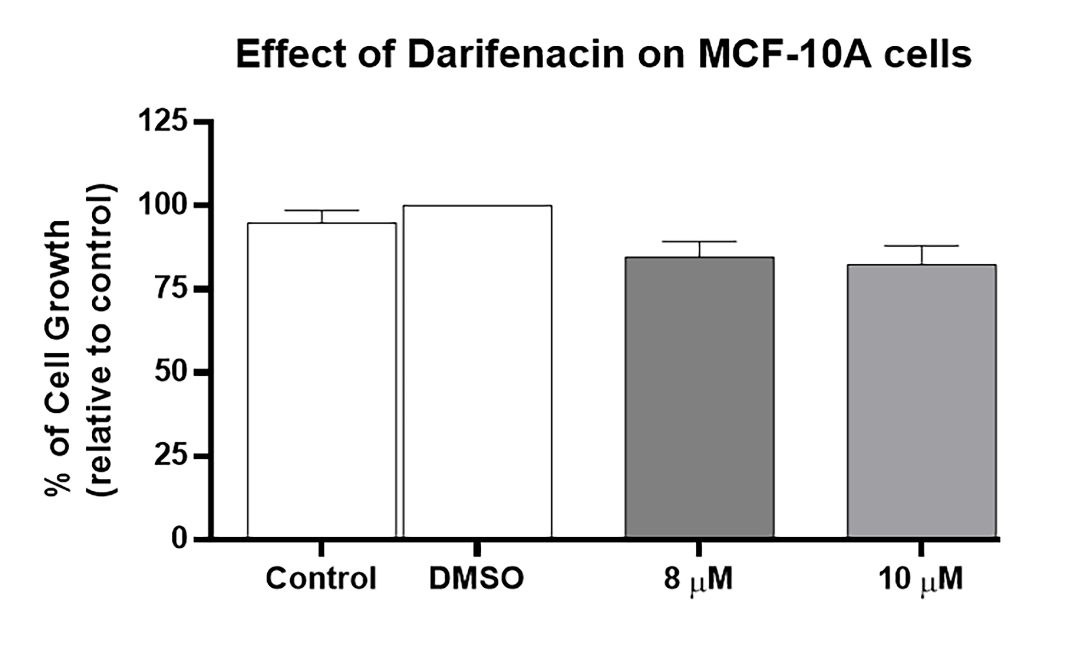
**

**Supplementary Figure 1.** Effect of darifenacin in MCF-10A non-tumorigenic cells, assessed by the SRB assay. Cells were treated for 48 h with darifenacin at 8 μM and 10 μM. The effect of the vehicle at the highest concentration tested was also evaluated. Results are presented as a % of cell growth and are the mean ± S.E.M. of at least three independent experiments.


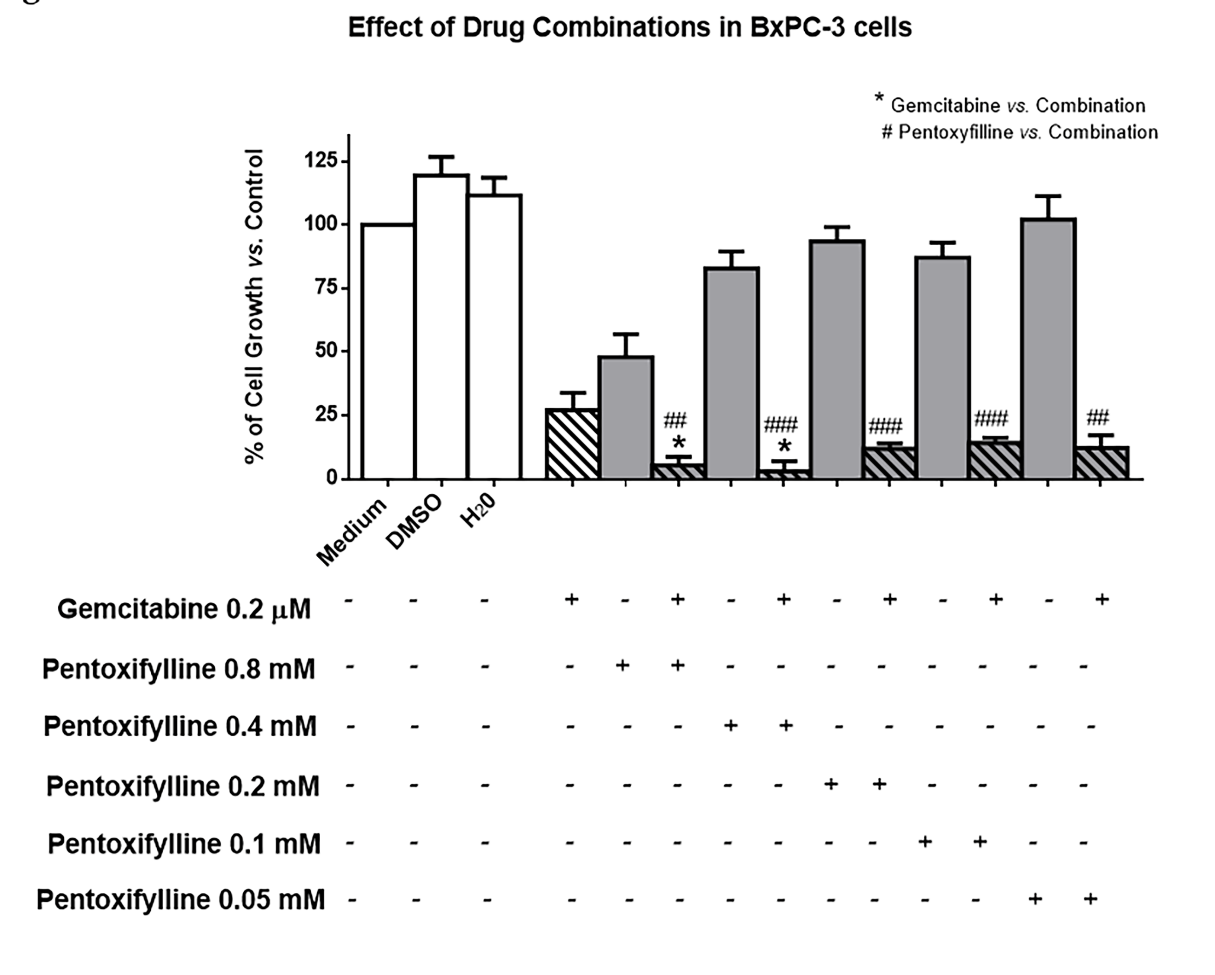


**Supplementary Figure 2.** Effect of the combined treatment of gemcitabine with pentoxifylline on the % of cell growth of BxPC3 pancreatic ductal adenocarcinoma (PDAC) cell lines, assessed by the SRB assay. Cells were treated for 48 h with drug combinations consisting of 0.2 μM gemcitabine with five serial dilutions of pentoxifylline (0.05 mM to 0.8 mM). The effect of the vehicle (DMSO) at the highest concentration tested was also analyzed. Results are presented as % of cell growth and are the mean ± S.E.M. of at least three independent experiments. * *p* < 0.05; ## *p* < 0.01; and ### *p* < 0.001.


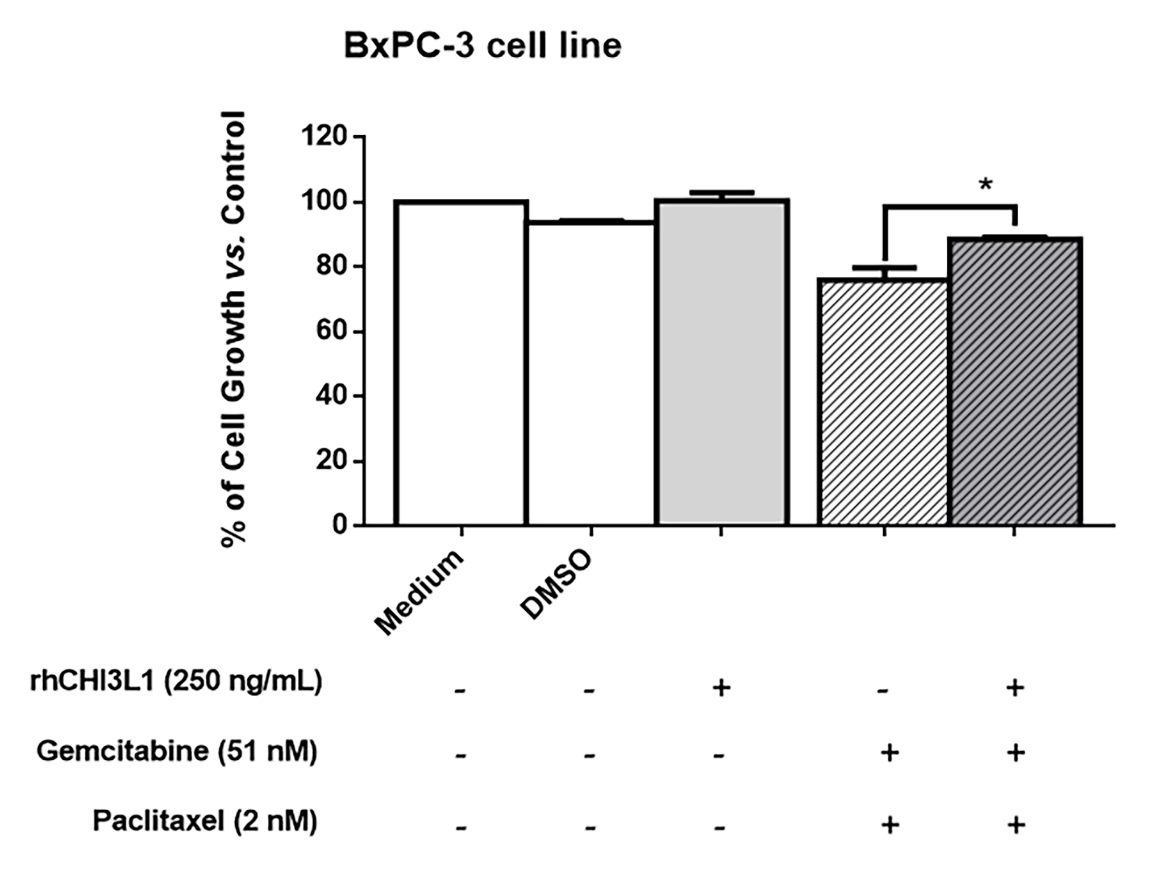


**Supplementary Figure 3.** Determination of the % of cell growth on BxPC-3 pancreatic ductal adenocarcinoma (PDAC) cells treated with gemcitabine and paclitaxel in the presence/absence of the recombinant human (rh) protein for CHI3L1 (rhCHI3L1). Cells were treated with gemcitabine at 51 nM and paclitaxel at 2 nM for 48 h, in the absence or presence of the rhCHI3L1 at 250 ng/mL, and cell growth was analyzed with the SRB assay. Results are the mean ± S.E.M. of at least three independent experiments. Analysis were performed by GraphPad using the Student t-test. * *p* < 0.05.


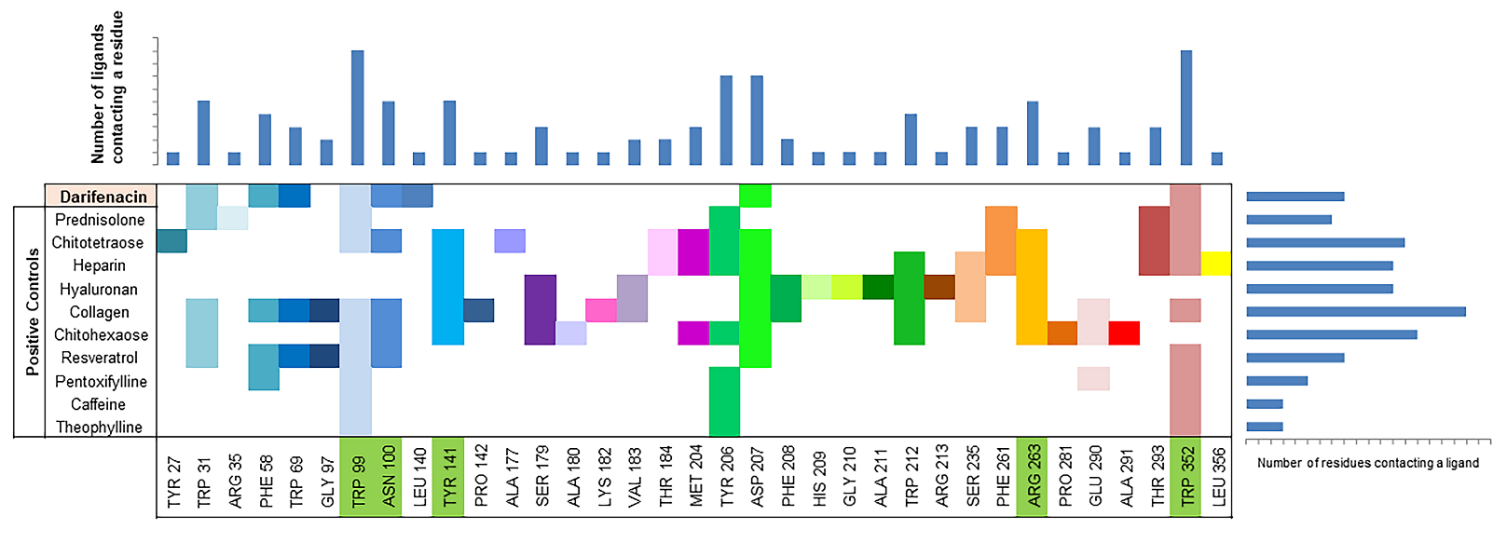


**Supplementary Figure 4.** Interaction histogram. Contact details of the positive controls and darifenacin with the binding pocket of CHI3L1. Residues involving the target CHI3L1 and a long chitin fragment are represented with a green background.

***Supplementary Tables***

**Supplementary Table 1.** GI_50_ concentration (nM) of paclitaxel in two pancreatic ductal adenocarcinoma (PDAC) cell lines.

|  | **Paclitaxel** |
| --- | --- |
| **PDAC cell lines** | **GI_50_ (nM)*** |
| **PANC-1** | 102.5 ± 4.4 |
| **BxPC-3** | 6.1 ± 0.9 |

* Concentration that causes 50% cell growth inhibition (GI_50_), determined 48 h following drug treatment, using the SRB assay. Results are the mean ± S.E.M. of at least three independent experiments.

**Supplementary Table 2.** Clinicopathological characteristics and correlation with mean overall survival (OS) of the PDAC patients. Abbreviations: OS, Overall Survival; Significant P-values are in bold.

| **Clinicopathological**  **characteristics** | **No,**  **%** | **OS months**  **(95% CI)** | ***P*** |
| --- | --- | --- | --- |
| **No. Patients** | 68 | 19.0 (16.3-21.7) |  |
| **Age, years** |  |  |  |
| **≤65** | 46 (67.6) | 20.2 (16.6-23.8) | 0.15 |
| **>65** | 22 (32.3) | 16.6 (12.9-20.2) |  |
| **Sex** |  |  |  |
| **Male** | 42 (61.8) | 17.5 (14.2-20.8) | 0.18 |
| **Female** | 26 (38.2) | 21.5 (16.9-26.0) |  |
| **Grading** |  |  |  |
| **1–2** | 33 (48.5) | 21.4 (17.0-25.7) | 0.09 |
| **3** | 35 (51.5) | 16.8 (13.7-20.0) |  |
| **Stage** |  |  |  |
| **IIA** | 12 (17.6) | 20.0 (12.6-27.4) | 0.83 |
| **IIB** | 54 (79.4) | 18.8 (15.9-21.7) |  |
| **CHRM3 expression** |  |  |  |
| **Low**  **High** | 34 (50)  34 (50) | 22.1 (17.8-26.4)  16.0 (13.1-19.0) | **0.017** |
|  |  |  |  |
